# Gambling Phenotypes in Online Sports Betting

**DOI:** 10.3389/fpsyt.2020.00482

**Published:** 2020-05-28

**Authors:** Roser Granero, Susana Jiménez-Murcia, Amparo del Pino-Gutiérrez, Bernat Mora, Eduardo Mendoza-Valenciano, Isabel Baenas-Soto, Mónica Gómez-Peña, Laura Moragas, Ester Codina, Hibai López-González, Teresa Mena-Moreno, Gemma Mestre-Bach, Susana Valero-Solís, Sandra Rivas, Zaida Agüera, Cristina Vintró-Alcaraz, María Lozano-Madrid, José M. Menchón, Fernando Fernández-Aranda

**Affiliations:** ^1^Department of Psychobiology and Methodology, Universitat Autònoma de Barcelona—UAB, Barcelona, Spain; ^2^Ciber Fisiopatología Obesidad y Nutrición (CIBERobn), Instituto Salud Carlos III, Madrid, Spain; ^3^Department of Psychiatry, Hospital Universitari de Bellvitge, L’Hospitalet de Llobregat, Barcelona, Spain; ^4^Psychiatry and Mental Health Group, Neuroscience Program, Institut d’Investigació Biomèdica de Bellvitge—IDIBELL, L’Hospitalet de Llobregat, Barcelona, Spain; ^5^Department of Clinical Sciences, School of Medicine, Universitat de Barcelona—UB, L’Hospitalet de Llobregat, Barcelona, Spain; ^6^Department of Public Health, Mental Health and Perinatal Nursing, School of Nursing, Universitat de Barcelona—UB, Barcelona, Spain; ^7^Ciber Salut Mental (CIBERsam), Instituto Salud Carlos III, Madrid, Spain

**Keywords:** clustering, gambling disorder, internet, online sports betting, phenotype

## Abstract

**Background and Objectives:**

The Internet provides easy access to multiple types of gambling and has led to changes in betting habits. A severe rise in problematic gambling has been predicted among all sectors of the population, and studies are required to assess the emerging phenotypes related to the new structures of gambling activities. This study aimed to explore the existence of latent classes associated with gambling habits among treatment-seeking gamblers due to Online Sports Betting (OSB).

**Method:**

Initial sample included *n* = 4,516 patients consecutively admitted for treatment in a hospital unit specialized in behavioral addictions. Two-step clustering analysis was used within the subsample of *n* = 323 patients who reported problems related with OSB, within a set of indicators including sociodemographics, psychopathological distress, personality, and severity of the gambling activity.

**Results:**

The prevalence of OSB as a main type of gambling problem in the study was 7.2% (95% confidence interval: 6.4 to 7.9%). Two latent clusters were identified, with differences in sociodemographics and clinical status. Cluster 1 (*n* = 247, 76.5%) grouped patients that were more affected due to the OSB behaviors, and it was characterized by non-married patients, lower socioeconomic position index, higher comorbidity with other substance related addictions, younger age, and early onset of the gambling activity, as well as higher debts due to the OSB, higher psychopathological distress, and a more dysfunctional personality profile. Cluster 2 (*n* = 76, 23.5%) grouped patients that were less affected by OSB, mostly married (or living with a stable partner), with higher social position levels, older age and older onset of the gambling activity, as well as a more functional psychopathological and personality profile.

**Conclusion:**

The increasing understanding of latent classes underlying OSB phenotypes is essential in guiding the development of reliable screening tools to identify individuals highly vulnerable to addictive behaviors among Internet gamblers, as well as in planning prevention and treatment initiatives focused on the precise profiles of these patients.

## Introduction

Despite the extensive research on the involvement in gambling related problems, a new phenomenon has recently emerged which is causing concern among specialists: Online Sports Betting (OSB). The expansion of this gambling modality in developed countries has significantly increased in parallel with opportunities to participate in online gambling services. Some experts advice that the characteristics of this betting modality could make it potentially more addictive and dangerous than other gambling activities, or even than betting at physical locations [online gambling sites are permanently accessible from anywhere there’s an Internet connection (24 hour-day, 7 day-week), gamblers can play *via* computer or mobile device at different sites (such as work, home), and online provides greater convenience, anonymity and comfort than other offsite platforms]. But although the potential unhealthy consequences associated with OSB, little research has been centered on this behavior. This study contributes to developing empirical knowledge regarding the phenotypes of the OSB, classically included as a subtype of the problematic or disordered gambling.

Gambling Disorder (GD) is a behavioral addiction involving a repeated and uncontrolled urge to gamble, with the consequence of clinical impairment or distress [DSM-5; (1)]. The DSM-5 taxonomy allows specifying the gambling severity level based on the number of criteria (mild, moderate, or severe gambling), while the ICD-11 (2) adds the subdivision into the subtypes of predominantly offline versus predominantly online.

Epidemiological studies regularly update worldwide prevalences for problem gambling, which estimates they were between 0.1% and 5.8% across five continents during the year before the survey, and between 0.7% and 6.5% during lifetime (3). The noteworthy increase in the incidences reported in meta-analytical data during the last decades has led to a large volume of studies aimed at increasing the understanding of the mechanisms explaining the onset and the progression of the GD (4). Much of the pioneer research in the gambling area had often contended that individuals who engage excessively in gambling behaviors experience a common set of symptoms, which are the result of shared risk factors, and which lead to similar treatment outcomes (5, 6). This assumption involved the grouping of different gambling types within a theoretical homogeneous condition, failing to take into account subtypes of gamblers based on how they engage in gambling activity and avoiding exploring how the mode of access could affect the latent phenotypes.

But in the recent years the gambling subtypes and the new emerging gambling modes have increased clinical and research interest, due to their supposed relevant role in the pathways of the gambling picture (7, 8). At present, multiple gambling modalities exist that differ in several aspects, such as the range of stakes involved, the odds of the winning, or the level of mental/physical skills required. The advances in technology and the universalization of Internet access have facilitated fast and easy access to almost all traditional manners of gambling globally. It has been observed that when gamblers can choose, they tend to select Internet instead of land-based modes, arguing, as the main reasons, convenience, higher fun-excitement-entertainment, greater comfort (online is accessible in their own homes), perception of greater capacity to win money, faster play speed, anonymity, and privacy (9). As a result, online gambling (also referred to as Internet, interactive, or remote gambling) is currently a particular area of interest, and many studies have emerged to investigate the characteristics and motivations of the growing population of Internet gamblers.

Online gambling does not represent a new gambling modality, since Internet is only a mode of access to multiple gambling platforms. Evidence suggests that the same activity experienced in online modalities versus venue/land-based forms may have particular features that can lead to different harms (10, 11). A relationship also exists, albeit complex, between the availability of the gambling activities and the level of the related problems (12). It seems that fast, easy, and constant access to gambling, as well as the ability to bet for uninterrupted periods in private settings, may contribute to the early onset of the gambling activities, and the high progression of the gambling related problems (10, 11). The particular structural characteristics and the interactive and immersive Internet environment could also adversely affect the gambling related harms (13). For example, the payment methods: compared to the cash procedure typical of many land/based games, the digital forms of money used through Internet system (e.g. credit cards, electronic funds transfers, or e-wallets) appear to lead to greater expenditures and increased gambling and losses (14). It has also been stated that online gambling environments contribute to problems with self-regulation and self-control on spending decisions (11, 15, 16), and that the rapid-sequential choice typical of computerized environments facilitates transactions and significantly contributes to gamblers’ decision to continue and intensify gambling behavior (17).

But the results on the differences between online and offsite gambling outcomes are still controversial. Some studies outline that adjusting by the gambling preference and other variables (such as the frequency of participation), the contribution of online access does not achieve the predictive capacity of the gambling impairment (18–20). It has also been observed that the dichotomy of online versus offline access to gambling is far too limited to appropriately understand differences in the subgroups of gamblers, and that considering the individuals’ life cycle by combining chronological age, socioeconomic position and marital status, should provide better insights into groups (21). It has also been postulated that it should be the mixed-mode of gambling (using both Internet and land-based modes, compared to internet-only and land-based only) that predicts the greatest overall involvement in gambling and the greatest level of gambling problems (22). Among clinical treatment-seeking samples, online pathological gamblers (compared to landed-based pathological gamblers) have shown limited differences, focused on slightly higher educational levels, higher socioeconomical positions, and larger amounts of bets and debts related to Internet forms (23). And even considering the legal status of Internet gambling (with great differences between countries), studies carried out across different European jurisdictions (ranging from prohibition of online gambling to broad legal access) have found no relevant differences in the prevalence rates of GD depending on the mode of access (24).

One of the most popular types of Internet gambling is OSB. It is heavily marketed and successfully targeted at the young adult male, with the consequence of hundreds of websites facilitating access to sportsbooks and fastest developing forms internationally. OSB represents an example of the potential interaction between the mode of access to the gambling activity (Internet versus land-based) and the presence and severity of the gambling related problems. One study has obtained a great difference in the prevalence of impairment related to the sports betting among Internet gamblers (67%) compared to land-based gamblers (23%) (25). Based on a large online survey, it has also been observed that, compared to non-Internet gamblers (both moderate-risk and problem gamblers), Internet gamblers that experience gambling-related harms are characterized by younger age individuals, engaged in a greater number of gambling behaviors, and more likely to bet on sports (26). Finally, it has been postulated that OSB gamblers (compared to non-sports Internet GD patients and GD patients who did not gamble online) represent a particular vulnerable subgroup characterized by distinct personality traits (higher persistence levels), riskier betting behavior, and higher debt levels (27).

But although there is cumulated evidence regarding OSB phenotype compared to other gambling types, most studies have been planned under the assumption that OSB constitutes a unique homogeneous phenotype, grouping mostly young, male, single, with medium to high education levels, employed, or full-time student (15). Although, lower income, minor ethnicity groups, immigrant situation, and engaging in multiple different gambling forms have also been reported as distinctive characteristics of the OSB profile (28). To the best of our knowledge no study has been conducted to identify latent empirical classes within OSB groups in a clinical treatment-seeking population.

### Objectives

To summarize, although there is increasing interest in online gambling, little has been reported in the scientific literature about the heterogeneity of the OSB profiles regarding demographic characteristics and other clinical features. This study aimed to explore the existence of empirical latent classes in a large sample of OSB treatment-seeking patients, using a broad set of indicator variables, including sociodemographics, problem gambling severity, psychological distress, and personality functioning. Based on the available empirical evidence obtained in different modes of gambling, we hypothesized that OSB constitute a mixed group in which latent underlying subgroups with different gambling profiles can be recognized. Since no previous study using this approach is available for OSB samples, we made no *a priori* assumptions about the number of expected groups.

Identifying the variables related to these empirical classes would facilitate the development of measurement tools with good discriminative ability and the planning of effective and precise prevention and treatment programs.

## Method

### Participants

The initial sample comprised *n* = 4,516 treatment-seeking patients consecutively attended to at the Pathological Gambling Unit and other Behavioral Addictions situated in the Bellvitge University Hospital (Barcelona), between January 2005 and August 2019. This hospital oversees the treatment of cases with behavioral addiction-related problems, and it is certified as a tertiary care center for the treatment of these psychiatric conditions. Data analyzed in the study corresponded to the first assessment before starting treatment. A total of 3,982 (88.2%) men were attended to, versus 534 women (11.8%). Most of the participants had achieved a primary or less (57.1%) education level, were single (41.9%) or married (44.8%), belonged to low socioeconomic levels (51.5%), and were employed (55%). The mean chronological age was 42.0 years old (SD = 13.9), and the mean duration of the gambling was 6.1 years (SD = 6.2). The most prevalent reason for seeking treatment in this behavioral addictions unit was GD (*n* = 3,987; 88.3%). The first block of **Table 1** contains the description of the initial complete sample.

**Table 1:** Characteristics of the patients in the study.

		Total sample (n = 4,516)				OSB subsample (n = 323)			
*Sociodemographics*		n		%		n		%	
Sex	Women	534		11.8		13		4.0	
	Men	3,982		88.2		310		96.0	
Education	Primary or less	2,577		57.1		106		32.8	
	Secondary	1,615		35.8		159		49.2	
	University	324		7.2		58		18.0	
Marital status	Single	1,892		41.9		197		61.0	
	Married-couple	2,023		44.8		99		30.6	
	Divorced-Separated	601		13.3		27		8.4	
Social status	High	65		1.4		10		3.1	
	Mean-high	224		5.0		34		10.5	
	Mean	484		10.7		49		15.2	
	Mean-low	1,418		31.4		126		39.0	
	Low	2,325		51.5		104		32.2	
Employment	Unemployed	2,032		45.0		106		32.8	
	Employed	2,484		55.0		217		67.2	
*Age and evolution*		*Min*	*Max*		*Median*	*Min*	*Max*		*Median*
Chronological age (yrs-old)		15	88		40	15	80		30
Onset of the addiction (yrs-old)		14	80		27	14	58		23
Duration of the addiction (yrs)		1	46		4	1	23		2

OSB, online sports betting; Min, minimum; Max, maximum.

A subsample of *n* = 323 patients who reported OSB related problems as the main reason for treatment-seeking was selected for exploring the existence of latent classes based on a set of indicators, including sociodemographic and clinical variables. The mean chronological age was 32.2 years old (SD = 9.7), and the mean duration of the betting behavior was 3.7 years (SD = 3.7). Most patients in this subsample were single (61.0%), employed (67.2%), and had achieved secondary education levels (49.2%). The second block of **Table 1** contains the description of the OSB subsample.

### Measures

*Diagnostic Questionnaire for Pathological Gambling* (according to DSM criteria) (29). This is a self-report questionnaire developed to identify the presence of GD using 19 items based on the DSM criteria [diagnoses are available for the DSM-IV-TR (30) and the DSM-5 versions (1)]. The psychometrical Spanish adaptation of this tool achieved adequate properties (Cronbach’s alpha α= 0.81 for a population-based sample and α=0.77 for a clinical sample) (31). The internal consistency achieved in this study was good (α=0.814).

*Symptom Checklist-Revised* (SCL-90-R) (32). This is a self-report questionnaire developed to assess the psychological state using 90 items factorized into nine primary (first order) dimensions (somatization, obsessive-compulsive, interpersonal sensitivity, depression, anxiety, hostility, phobic anxiety, paranoid ideation, and psychoticism), and three global indices [global severity index (GSI), total positive symptoms (PST), and positive symptoms discomfort index (PSDI)]. The psychometrical Spanish adaptation of this tool obtained adequate properties (the mean Cronbach’s alpha was α = 0.75) (33). The internal consistency in our sample was also in the adequate to good range (α = 0.790, for the paranoid ideation scale, to α = 0.981 for the global indices).

*Temperament and Character Inventory-Revised* (TCI-R) (34). This is a self-report questionnaire developed to assess personality traits using 240 items based on the Cloninger’s multidimensional model, and structured into seven factors [four for temperament (novelty seeking, harm avoidance, reward dependence, and persistence), and three for character (self-directedness, cooperation, and self-transcendence)]. The psychometrical Spanish adaptation of the tool obtained adequate properties (the mean Cronbach’s alpha was α = 0.87) (35). The internal consistency in the sample of the study was in the adequate to good range (α = 0.703, for novelty seeking, to α = 0.868 for persistence).

*Other variables*. This study also analyzed additional data assessed using a semi-structured interview. This tool covered socio-demographic characteristics (sex, marital status, education level, employment status), as well as the socio-economic position index, according to Hollingshead’s scale (based on the participants’ level of education and profession) (36). Patients also completed questions related to OSB problem-related variables (age of onset, duration, bets per gambling/episode, and cumulated debts due to the gambling addiction) and substance use (no vs. at least sometimes).

### Procedure

The study was carried out in accordance with the Declaration of Helsinki principles, and approved by the Ethics Committee of University Hospital of Bellvitge (Barcelona). All patients provided signed informed consent for participating in the research. There was no financial or other compensation for being part of the sample of the study.

The assessment process took place in a single session lasting about 90 min. Data for the semi-structured interview were collected by psychologists and psychiatrists with high experience in the treatment of behavioral addictions. The clinicians also helped participants to complete the self-report questionnaires in order to guarantee that no data were missing (for example, due the lack of understanding).

### Statistical Analysis

The statistical analysis was carried out with SPSS24 for windows (37). The identification of the latent empirical classes was based on a two-step cluster procedure, a method used to explore the existence of natural groupings within a dataset of categorical and continuous variables. This method uses an agglomerative hierarchical clustering system and allows automatic determination of the optimal number of groups. This study used the log-likelihood distance and the Schwarz Bayesian Information Criterion (BIC), and Akaike’s Information Criterion (AIC) to determine the best model (the optimal number of latent classes was considered for the model with the largest ratio of changes for the BIC and AIC, as well as the largest ratio of distances measured comparing the current number of clusters against the previous number).

The indicator variables in the two-step clustering included sociodemographic features (sex, marital status, and social position index), global psychopathological distress (SCL-90R GSI), personality profile (TCI-R scales), OSB severity (number of the DSM-5 criteria for gambling), and substances use. The quality of the clustering was measured using the Silhouette index, a cohesion-separation measurement interpreted as how similar individuals are to their own cluster compared to other clusters) (38). Silhouette values are into the range −1 to +1, and high values are indicative of adequate matching in one’s own cluster and of poor matching in other clusters (values lower than 0.30 are interpreted as poor fits, between 0.30 and 0.50 as fair, and higher than 0.50 as good).

The comparison between the latent empirical clusters was based on Chi-square tests (χ^2^) for categorical variables and on *t*-Test procedures for quantitative measurements. The effect sizes for the proportion and mean differences were based on the standardized Cohen’s-*d* coefficient, considering poor-low effect size for |*d*| > 0.20, moderate-medium for |*d*| > 0.5, and large-high for |*d*| > 0.80 (39). The increase in the Type-I errors due to the multiple statistical tests for comparing the clusters was controlled with the Finner method (included in the stepwise familywise error rate procedures) (40).

## Results

### Prevalence of OSB in the Study

**Figure 1:**
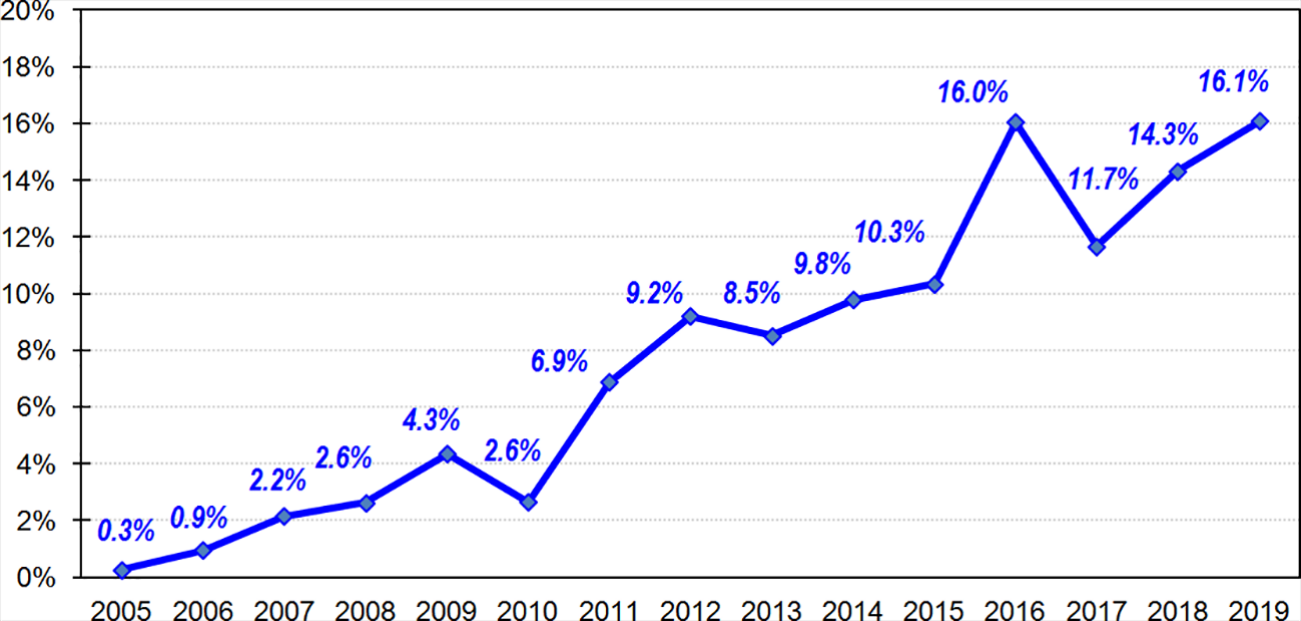
Prevalence of the prevalence of consultation due to OSB during the recruitment of data (n=4,516).

Within the initial complete sample (N = 4,516), the number of patients who reported OSB as primary or secondary reason for treatment-seeking was *n* = 323 [prevalence = 7.2%; 95% confidence interval (95%CI): 6.4 to 7.9%]. **Figure 1** shows the line-plot for the prevalence of consultations due to OSB during the recruitment of the data in the study (obtained for the complete sample, N = 4,516). This plot displays an upward trend over time, with rates of 0.3% during 2005 to 16.1% during 2019.

OSB was the reason for treatment-seeking in *n* = 323 patients (prevalence = 7.2%; 95%CI: 6.4 to 7.9%). A number of *n* = 230 patients reported the presence of OSB concurrent with other secondary comorbid forms of gambling or behavioral addiction [prevalence within the total sample: 5.1% (95%CI: 4.5 to 5.7%); prevalence within the OSB subsample: 71.2% (95%CI: 66.3 to 76.1%)]. The number of OSB patients with substance use was *n* = 161 [prevalence within the total sample: 3.6% (95%CI: 3.0 to 4.1%); prevalence within the OSB subsample: 49.8% (95%CI: 44.4 to 55.3%)]. **Table S1** (**Supplementary Material**) contains the distribution of the secondary comorbid forms of gambling and behavioral addictions within the OSB subsample, as well as the substances use.

### Clustering Procedure

**Table S2** (**Supplementary Material**) shows the results of the auto-clustering in the OSB subsample. The optimal number of clusters chosen by the system was two: it achieved the largest ratio changes for BIC and AIC (1.00 in both estimations), the highest ratio distance (1.60) and the highest cohesion/separation measurement (Silhouette = 0.30, into the fair range). Since this two-cluster model also achieved good clinical interpretation, it was selected as the best.

**Figure 2:**
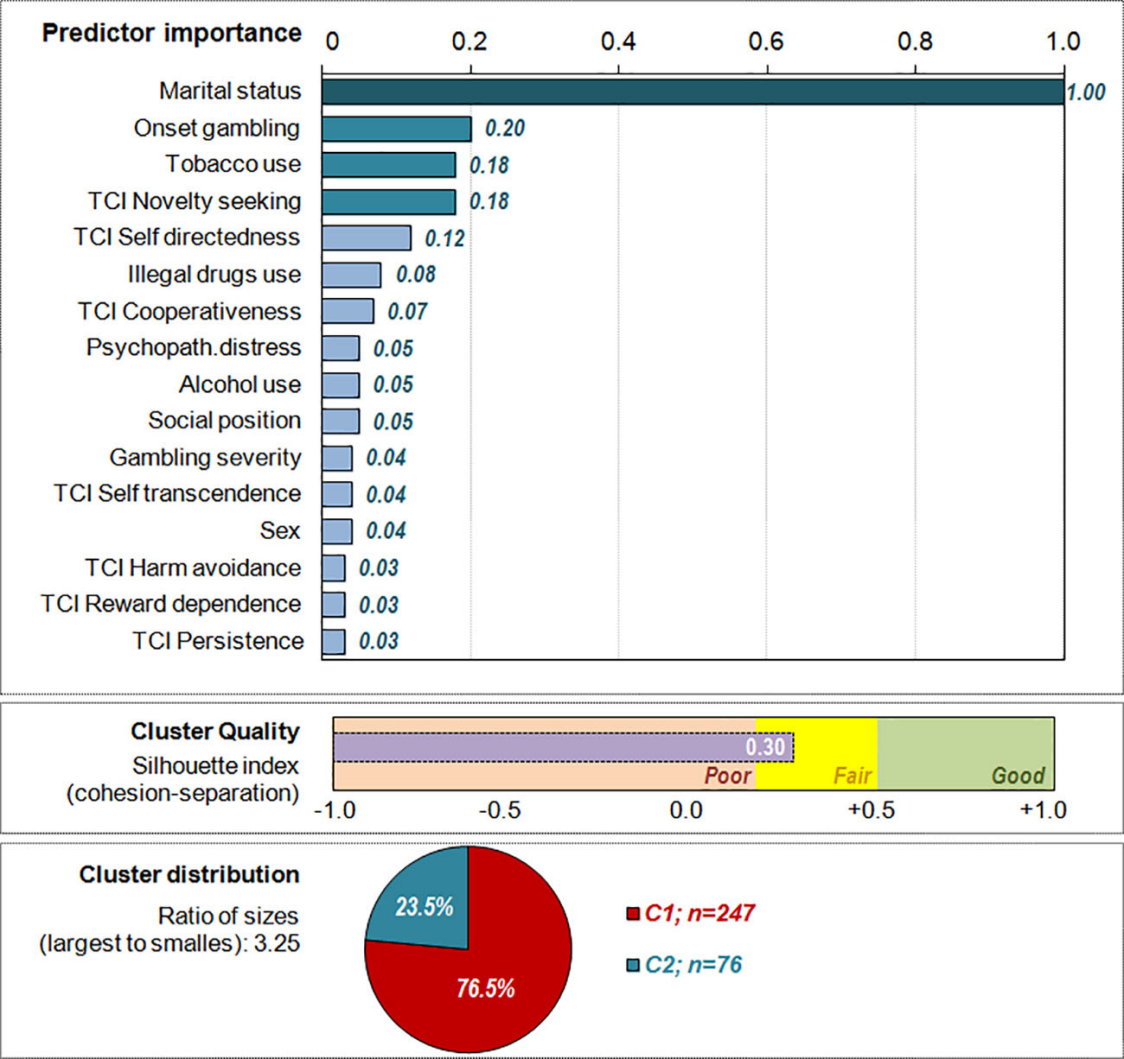
Results of the clustering procedure within the sports betting online subsample (n = 323).

The first panel in **Figure 2** shows the ordered bar-chart with the relative predictor importance in the clustering, and provides a measure of the discriminative capacity of each variable. Relative relevance is reported in a range 0 to 1: the greater the relevance of the indicator, the less likely it is that changes between clusters for the variable are attributable to chance. The predictor with the greatest significance in the study was the marital status, followed by the onset of the gambling, the novelty seeking score, tobacco use, and novelty seeking (these variables are plotted with the darkest color bars). The remaining predictors achieved lower significance, as they were the variables with the poorest contribution to harm avoidance, reward dependence, and persistence levels. The second panel contains the graphic representation of the Silhouette index in the study, and the third panel the cluster distribution (the ratio of sizes was 3.25, since cluster 1 achieved nearly one third of the OSB subsample).

### Comparison Between the Latent Empirical Clusters

**Table 2** shows the comparison between the empirical clusters identified in the study. Cluster 1 grouped *n* = 247 patients, which represented 76.5% of the OSB patients. This latent cluster was characterized by unmarried marital status, poorer socioeconomic levels, higher prevalence of substances use (tobacco, alcohol, and other illicit drugs), younger age, early onset of the OSB activity, higher severity of the betting activity, higher psychopathological distress and more dysfunctional personality profile (higher scores in novelty seeking and lower levels in self-directedness and cooperativeness). Cluster 2 grouped the remaining *n* = 76 patients (23.5% of the OSB subsample), and it was characterized by married marital status (or living with a stable partner), higher social position levels, older age and older onset of the OSB activity, lower severity associated with the OSB, and a more functional psychopathological and personality profile. The prevalence of substance use was also lower.

**Table 2:** Comparison between the latent clusters identified within the OSB subsample.

		Cluster 1 (n = 247; 76.5%)		Cluster 2 (n = 76; 23.5%)		p	|d|
		n	%	n	%		
Sex	Women	12	4.9	1	1.3	.169	0.21
	Men	235	95.1	75	98.7		
Education	Primary or less	76	30.8	30	39.5	.150	0.18
	Secondary	129	52.2	30	39.5		0.26
	University	42	17.0	16	21.1		0.10
Marital status	Single	197	79.8	0	0.0	.001*	**2.21^†^**
	Married-couple	26	10.5	73	96.1		**2.08^†^**
	Divorced-Separated	24	9.7	3	3.9		0.23
Social position index	High	6	2.4	4	5.3	.012*	0.15
	Mean-high	25	10.1	9	11.8		0.06
	Mean	29	11.7	20	26.3		0.38
	Mean-low	103	41.7	23	30.3		0.24
	Low	84	34.0	20	26.3		0.17
Employment	Unemployed	90	36.4	16	21.1	.012*	0.34
	Employed	157	63.6	60	78.9		
Other behavioral addictions		177	71.7	53	69.7	.746	0.04
Tobacco		133	53.8	13	17.1	< .001*	**0.80^†^**
Alcohol		29	11.7	1	1.3	.006*	**0.52^†^**
Other drugs		36	14.6	0	0.0	< .001*	**0.78^†^**
		*Mean*	*SD*	*Mean*	*SD*	*p*	*|d|*
Chronological age (yrs-old)		30.41	9.33	37.95	8.76	< .001*	**0.83^†^**
Age of onset of gambling (yrs-old)		23.66	6.91	29.58	8.22	< .001*	**0.78^†^**
Duration of the addiction (yrs)		3.89	3.76	3.15	3.27	.125	0.21
Number of DSM-5 criteria		7.32	1.79	6.82	1.85	.032*	0.28
^1^Maximum bets (euros-episode)		800	1700	700	1775	.795	0.03
^1^Mean bets (euros-episode)		40	140	35	190	.430	0.10
^1^Debts due to the OSB		6500	20250	2300	14000	.023*	0.23
SCL-90R GSI		1.07	0.69	0.82	0.55	.005*	0.40
Novelty seeking		113.91	13.43	103.64	13.39	< .001*	**0.77^†^**
Harm avoidance		99.96	17.21	97.55	14.52	.270	0.15
Reward dependence		96.54	14.21	98.57	13.87	.276	0.14
Persistence		106.30	20.84	105.84	18.26	.863	0.02
Self-directedness		126.27	21.59	139.21	21.59	< .001*	**0.60^†^**
Cooperativeness		126.62	17.95	134.37	14.14	< .001*	0.48
Self-transcendence		60.39	14.57	57.38	13.55	.111	0.21

OSB, online sports betting; SD, standard deviation.

^1^Median and interquartile range.

*Bold: significant comparison.

^†^Bold: effect size into the mild-moderate (|d| > 0.50) to large-high range (|d| > 0.80).

**Figure 3:**
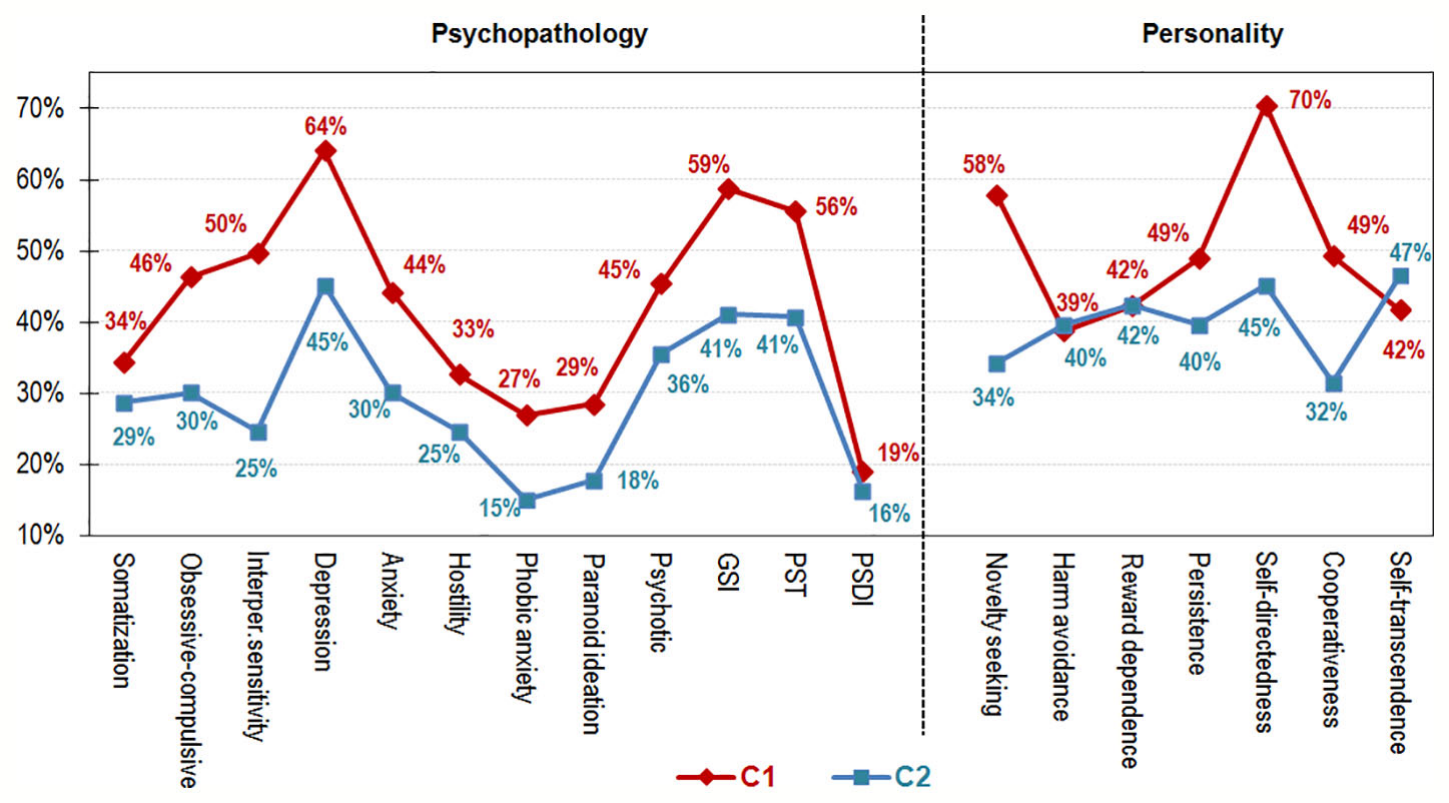
Line-chart within the sports betting online subsample (n = 323).

**Figure 4:**
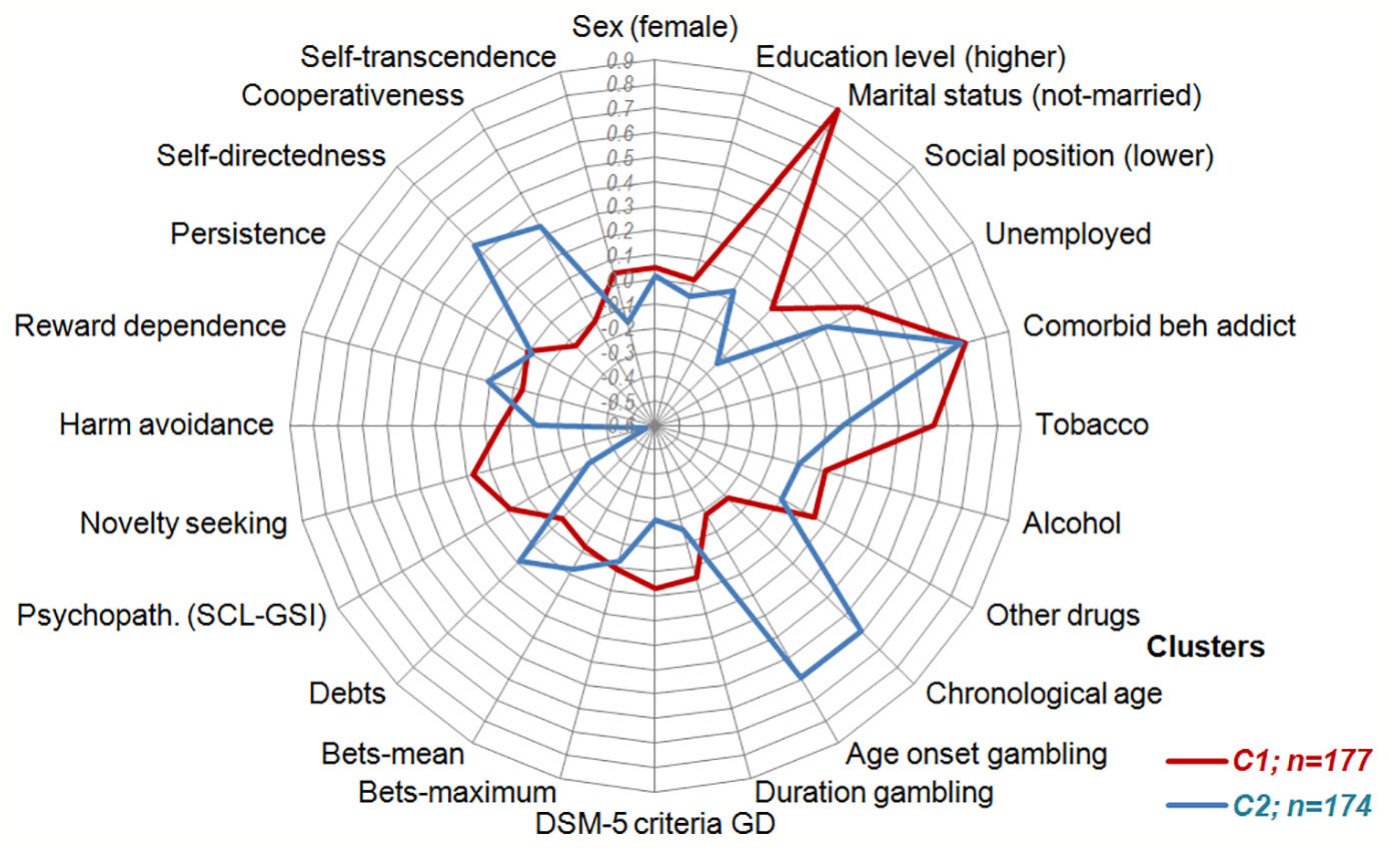
Radar-chart within the sports betting online subsample (n = 323).

**Figures 3** and **4** graphically show the results of the comparison between the clusters, which contribute to the understanding of the two latent classes within the OSB patients. **Figure 3** displays the line-chart with the prevalence of patients outside the normal range in the SCL-90R (psychopathological state) and the TCI-R scales (personality traits). As a whole, compared to Cluster 2 a higher percentage of patients within Cluster 1 reported mean scores within the clinical range in the SCL-90R, being the highest impairing level in the depression scale and in the global indexes GSI and PST; regarding the personality traits, the highest deviances from normal ranges were for self-directedness and novelty seeking. **Figure 4** displays the radar-plot with the main variables analyzed in the study (proportions area plotted for categorical variables and z-standardized means for quantitative variables, to allow easy interpretation due the difference in the metric scale of the variables). As a whole, Cluster 1 represented the profile of OSB patients that are more affected, while Cluster 2 represented the profile of those less affected.

## Discussion

The current study estimated the prevalence of the OSB among a large clinical sample of patients attended in a hospital unit specialized in the treatment of GD and other behavioral addictions. The clustering analysis then examined the variance within the OSB subsample, with the aim of identifying latent homogeneous subgroups. The phenotypical differences between the two empirical clusters of OSB as regards sociodemographics, gambling severity, psychopathological state, and personality, provided reliability and validity to the clustering.

Earlier studies fueled concerns that online gambling significantly contributes to the onset and progression of the gambling related problems, with the result of prompting research into the characteristics and associated risks of this mode of access to gambling activities (7, 41). But while the insights provided by these studies into the profiles of problem gamblers that gamble online, they did not account for the relevant issue of the heterogeneity and within-subjects variance among the samples. This paper is the first, to our knowledge, to explore latent subgroups in a clinical sample of OSB patients. The rationales of this study were: a) the assumption that not all problematic online gamblers form a homogeneous group with common features and shared vulnerabilities, and therefore to automatically attribute the global Internet gambling habits and traits amongst OSB patients is inaccurate; and b) the requisite to distinguish modes of gambling (online versus land-based) and gambling forms (e.g., slot-machines, lottery, sports betting, …) to adequately characterize gambling related profiles, as well as more specific and personalized treatment approaches for each type of patient.

The clustering analysis in this work revealed that two distinctive latent subgroups was the optimal grouping solution for the study that, respectively, represented latent phenotypes of OSB. The characterization of these subgroups seemed to suggest a dimensional factor varying in the psychological and functional affectation. This result is in line with previous studies, which have published a higher likelihood for experiencing psychological distress among problematic online gamblers compared to non-problematic online gamblers (42). To explain these results, it has been argued that online access to gambling may become particularly motivating for escaping and alleviating negative emotional states, since Internet provides privacy, is less socially demanding than many land-based gambling activities, and allows gambling sessions without distractions and interruptions. This results in a vicious circle: individuals with high risk of negative mood and anxiety states should find in online gambling an easy way to escape and control emotional distress (43), but the higher the immersion in online gambling activity the higher are the increases in the gambling harms and their correlates (including the general psychopathological state).

Associated with the worse mental state among the patients within the cluster with the higher affectation, this latent subgroup also reported higher comorbidity with substance use (tobacco, alcohol, and other drugs). In fact, the tobacco consumption was one predictor with high importance in the clustering, after the marital status and the onset of the OSB activity. This finding is also consistent with previous studies, which have related a higher likelihood of substance use while gambling (mainly for alcohol and illicit drugs) among people with online gambling habits (44). Increased prevalences of substance-related disorders among Internet problematic gamblers compared with other gambling forms and with non-gamblers have also been reported (45). Epidemiological and etiological research has also shown the relevance of the co-occurrence between online gambling and substances consumption (mainly with tobacco), as well as the relationship between substances status with more severe gambling habits (46). Our results, together with this set of findings, should again suggest that the solitary and private settings allowed by Internet gambling may undermine rational decision-making and increase the ease of substance use. Online access at home may also facilitate gambling alone at any time of the day for long continuous sessions, and these contextual features should make substances more likely to be consumed. Future research should assess how gambling alone, timing, and duration of online play contribute to the gambling severity among OSB.

As regards the comorbid concurrence of OSB with other addictive behaviors, previous studies have shown a strong relationship between online gambling and engaging in a wide range of other behavioral addictions (land-based and online activities) (47). In our study, both latent empirical clusters achieved high prevalences of comorbid forms of gambling and/or other behavioral addictions (71.7% and 69.7%, with a low difference of 2.0% between the classes). The strong links (co-occurrences) within addictions obtained in the scientific studies have suggested the notion that some people are more prone to these problems, regardless of whether these involve substances or other behavioral activities (48). This higher vulnerability has been explained on the basis of a spectrum that grouped a number of disorders drawn from several diagnostic categories that share core impulsive-compulsive features. This construct has been supported by many studies (49–51), who have placed GD, substance use disorders and other behavioral addictions (sex, buying or gaming) toward the upper band of the impulsive trait in the spectrum (the opposite upper band of the compulsive trait in the spectrum included disorders such as obsessive-compulsive, body dysmorphic or restrictive-type anorexia nervosa). This theoretical assumption could explain the results of our study, which showed a joint association between the presence of OSB and the high likelihood of other multiple addictions.

Regarding personality traits, the higher affectation cluster was characterized by a more dysfunctional profile defined by higher scores in novelty seeking and lower scores in self-directedness and cooperativeness. Novelty seeking is a personality trait strongly related with the exploratory level of the individuals in response to novel situation and the impulsive decision making (52), and it has been considered in research and clinical settings as a measure of the individuals’ (in)capacity to bring responses into standards and to support the pursuit of long-term goals and as a consequence a powerful risk factor for psychopathology (53, 54). High levels on the novelty seeking dimension has been linked to all stages of addictions, from the acquisition phase of a single addiction to the escalation to multiple concurrent addictive behaviors (51, 55, 56). This characteristic has also been systematically obtained by comparing problematic and disordered gamblers with non-gamblers controls, and it has also been identified as a strong predictor of the gambling severity (57). Self-directness is the ability to adapt-regulate owns’ behavior to the demands of a situation in order to achieve personally chosen goals and values, while cooperativeness is described as the capacity of the individuals for being empathic, helpful, socially tolerant and compassionate. Adequate functioning in self-directedness and cooperativeness seem play a relevant role in fast and adaptive emotional responses and in the choice of cognitive regulation strategies (58), and its relevance in the stress response has also been consistently reported (59). Along this line, some researchers have suggested that a combination of both low self-directedness and low cooperativeness could form a general factor representing low psychological maturity, a temperamental vulnerability predictive of many psychiatric disorders (60, 61). This intrinsic aspect of the global mental health has also been interpreted as an epiphenomenon, a “marker” of the neuropsychiatric dysfunctions in individuals who show a lack of sense of responsibility, self-control, and social skills, which ultimately are part of the definitions of the addictive disorders (substance and non-substance) (62). Finally, our results regarding the personality profile related to the more affected cluster (higher novelty seeking score and lower self-directedness and cooperativeness) are consistent with previous studies which reported the contribution of these domains on the gambling area (63–65). A recent path-analysis study has also observed that at a young age, the combination of a profile defined by immature character (low self-directedness and cooperativeness) with extreme temperament (high novelty seeking) may be a predictor of substance addiction across sex (with direct and indirect effects on the mental health status) (66). Future research should analyze the longitudinal predictive capacity of this particular endophenotype on the onset and progression of the OSB related problems.

Previous research into the OSB area have also emphasized distinguishing demographic features for this gambling subtype, such as the individuals’ male sex, young age, not-married marital status (mostly single), medium or higher education levels and employed as full-time student (11, 47). The results of this study are in line with these findings. The description of the OSB subsample revealed that most patients were male (96%), with secondary or university education levels (67.2%), unmarried (69.4%), and employed (67.2%). The mean of the chronological age among OSB subsample was also younger compared to the mean obtained among the total sample (32.2 versus 42.0 years-old), as well as the age of onset of the addictive gambling (25.1 versus 29.6 years-old), and the progression of the disorder (3.7 versus 6.1 years). The results obtained in the OSB clustering reinforced these findings as regards the contribution of the sociodemographic features in the variability of the phenotype. The higher affectation latent group was related to more deprived social positions, unemployed situation, unmarried marital status, younger age, and early age of onset of the gambling activity. Other studies have also showed a relationship between lower income and the severity of the problematic online gambling (67), and being divorced/separated or living common-law has been described as the marital status most predictive of the worse harms related to the Internet gambling (68). Lastly, it should be emphasized that research studies focused on specific populations highly vulnerable to gambling problems have found that unemployed/low income/poverty, unmarried status, young age, and early age are powerful risk factors for experiencing the greatest severe consequences of the gambling activity, independent of the gambling forms/types and the online versus offline access (69).

Finally, our results are also consistent with previous research focused on the identification of separate profiles among sports betting. The secondary data analysis published by LaBrie and Shaffer on a longitudinal study among a large sample of subscribers to an Internet sports gambling site observed, using discriminant function analysis, a particular subgroup of individuals that made more and larger bets, bet more frequently, and were more likely to exhibit intense betting soon after enrollment (70). The results of our study, obtained in a clinical sample of treatment-seeking patients, adds empirical evidence about the heterogeneity of the gambling habit profiles among OSB, with a worse psychopathological state (higher distress and comorbid patterns with substance and non-substance behaviors), a higher affectation latent subgroup, also characterized also by a higher duration of the addiction patterns, higher debts related to the gambling activity, and personality traits defined by higher scores in novelty seeking, and lower self-directedness and cooperativeness.

### Limitations and Strengths

Some limitations should be considered when interpreting the results of this study. Firstly, the analysis of cross-sectional data restricts the temporal analysis of causative associations, and future longitudinal studies should explore the predictive capacity of the identified phenotypes (for example in treatment outcomes or developmental trajectories of the gambling problem). Secondly, the low prevalence of women within the OSB subsample restricts generalization of the findings, and makes results potentially non-representative of the female population with OSB. It should be considered that the frequency of women in the study is consistent with the point prevalence estimates in clinical treatment-seeking samples in the gambling area (GD is highly more frequent among men). We decided retaining the women in the study to increase the ecological validity of the study, and to be able to provide pioneer results in the area for OSB women.

A strength of the current study is the use of clustering procedure to identify the latent empirical groups among OSB patients, based on a relatively large set of predictors, including sociodemographics and clinical features. Compared with usual analytical procedures, cluster analysis does not require *a priori* assumptions regarding the underlying profiles in the sample, and therefore it allows empirically identifying the systematic covariation of multiple features contributing to the inter-individual variance in the gambling habits. A second strength is the relatively large sample size for the two latent subgroups identified (247 and 76 patients), which suggest that the clusters adequately cover the variance of naturally occurring individual differences (likelihood of small extreme groups are minimized). The third strength is the assessment of other behavioral and substance related addictions different to OSB. The high comorbidity rates found in our study warn of the high vulnerability of the patients for the concurrent presence of multiple addictive problems and the need of early screening tools and prevention plans.

### Conclusion and Implications

In conclusion, Internet gambling has become a relative newcomer to the world of gambling opportunities. The amount (in number and variety) of online applications has progressed with hasty speed during the last decades, offering changes and increases in sports betting opportunities. This study is the first to systematically analyze individual variance of OSB in a large clinical sample, and the clusters obtained provides empirical evidence about the existence of different latent phenotypes associated with the Internet sports betting habits. The identification of a latent subgroup of patients with higher affectation could suggest that OSB may be largely attractive for some highly vulnerable individuals, who can isolate and immerse in these activities in their home environment as an “escape problem” way, with the consequence of increased harms. The results of this work sought to provide a more accurate assessment of these patients in whom the gambling problems stemmed from OSB, as well as to identify highly vulnerable individuals from the general population. The findings of this work should also prove useful for planning effective prevention programs for developing effectiveness intervention therapies focused on the needs of the patients.

## Data Availability Statement

The datasets generated for this study will not be made publicly available because the data used in this study is part of the hospital database, and it is restricted to protect patients’ confidentiality.

## Ethics Statement

This study was carried out in accordance with the latest version of the Declaration of Helsinki principles. The Ethics Committee of the Bellvitge University Hospital approved the study and written informed consent was obtained and signed from all final participants.

## Author Contributions

Conceptualization: SJ-M, FF-A. Data curation: AP-G. Statistical analysis: RG. Investigation: BM, EM-V, IB-S, MG-P, LM, EC, HL-G, TM-M, GM-B, SV-S, SR, CV-A, ML-M. Project administration: SJ-M, FF-A, JM. Resources: AP-G, ZA. Writing of the first draft: SJ-M, RG. Review and critique: FF-A, JM.

## Conflict of Interest

The authors declare that the research was conducted in the absence of any commercial or financial relationships that could be construed as a potential conflict of interest.
