# Supplementary material for: Gambling Phenotypes in Online Sports Betting
Source: Front Psychiatry. 2020 May 28;11:482. doi: 10.3389/fpsyt.2020.00482 (PMC7270333; doi:10.3389/fpsyt.2020.00482)
Supplement: Supplementary file 1 [file Table_1.docx]

Supplementary Material

# Supplementary Tables

***Table S1 (Supplementary)*** *Prevalence of OSB in the study*

| **Total sample (*n*=4,516)** | ***n*** | ***Prev.%*** | ***95%CI*** | |
| --- | --- | --- | --- | --- |
| Online sports betting: primary gambling preference | 323 | 7.2% | 6.4% | 7.9% |
| OSB: without other forms of gambling or behavioral addictions | 93 | 2.1% | 1.6% | 2.5% |
| OSB: with other forms of gambling or behavioral addictions | 230 | 5.1% | 4.5% | 5.7% |
| OSB: without substances use | 162 | 3.6% | 3.0% | 4.1% |
| OSB: with substances use | 161 | 3.6% | 3.0% | 4.1% |
| **Online sports betting subsample (*n*=323)** | ***n*** | ***Prev.%*** | ***95%CI*** | |
| OSB: without other forms of gambling or behavioral addictions | 93 | 28.8% | 23.9% | 33.7% |
| OSB: with other forms of gambling or behavioral addictions | 230 | 71.2% | 66.3% | 76.1% |
| Other secondary forms of gambling |  |  |  |  |
| Slot-machines | 69 | 21.4% | 16.9% | 25.8% |
| Betting on horses | 67 | 20.7% | 16.3% | 25.2% |
| Casino | 54 | 16.7% | 12.6% | 20.8% |
| Internet gaming | 26 | 8.0% | 5.1% | 11.0% |
| Betting on cards | 23 | 7.1% | 4.3% | 9.9% |
| Lotteries | 22 | 6.8% | 4.1% | 9.6% |
| Gambling offices | 15 | 4.6% | 2.3% | 6.9% |
| Bingo | 13 | 4.0% | 1.9% | 6.2% |
| Football pools | 5 | 1.5% | 0.2% | 2.9% |
| Stock market | 1 | 0.3% | 0.0% | 0.9% |
| Other secondary behavioral addictions |  |  |  |  |
| Other new technologies problems | 21 | 6.5% | 3.8% | 9.2% |
| Videogames addiction | 11 | 3.4% | 1.4% | 5.4% |
| Compulsive buying | 3 | 0.9% | 0.0% | 2.0% |
| **Online sports betting subsample (*n*=323)** | ***n*** | ***Prev.%*** | ***95%CI*** | |
| Without substances use-abuse | 162 | 50.2% | 44.7% | 55.6% |
| With substances use-abuse | 161 | 49.8% | 44.4% | 55.3% |
| Tobacco use | 146 | 45.2% | 39.8% | 50.6% |
| Alcohol use-abuse | 30 | 9.3% | 6.1% | 12.5% |
| Other drugs use-abuse | 36 | 11.1% | 7.7% | 14.6% |

*Note.* OSB: online sports betting. 95%CI: 95% confidence interval.

***Table S2 (Supplementary)*** *Results of the auto-clustering*

| #  Clust. | BIC | ^a^BIC  Change | ^b^Ratio BIC  Changes | AIC | ^a^AIC  Change | ^b^Ratio AIC Changes | ^c^Ratio Distance | Silohuette | |
| --- | --- | --- | --- | --- | --- | --- | --- | --- | --- |
| 1 | 4829.82 |  |  | 4829.82 |  |  |  |  |  |
| 2 | 4656.84 | -172.98 | 1.00 | 4656.84 | -172.98 | 1.00 | 1.60 | 0.30 | (fair) |
| 3 | 4505.34 | -151.50 | 0.88 | 4505.34 | -151.50 | 0.88 | 1.07 | 0.20 | (poor) |
| 4 | 4476.02 | -29.33 | 0.17 | 4476.02 | -29.33 | 0.17 | 1.13 | 0.20 | (poor) |
| 5 | 4470.77 | -5.24 | 0.03 | 4470.77 | -5.24 | 0.03 | 1.45 | 0.10 | (poor) |
| 6 | 4520.80 | 50.02 | -0.29 | 4520.80 | 50.02 | -0.29 | 1.05 | 0.10 | (poor) |
| 7 | 4577.24 | 56.44 | -0.33 | 4577.24 | 56.44 | -0.33 | 1.05 | 0.10 | (poor) |
| 8 | 4638.84 | 61.60 | -0.36 | 4638.84 | 61.60 | -0.36 | 1.06 | 0.10 | (poor) |
| 9 | 4706.57 | 67.73 | -0.39 | 4706.57 | 67.73 | -0.39 | 1.20 | 0.10 | (poor) |
| 10 | 4792.18 | 85.61 | -0.49 | 4792.18 | 85.61 | -0.49 | 1.06 | 0.10 | (poor) |

*Note.* #Clust: number of clusters. BIC: Schwarz's Bayesian Criterion; AIC: Akaike's Information Criterion.

^a^The changes are from the previous number of clusters in the table.

^b^The ratios of changes are relative to the change for the two cluster solution.

^c^The ratios of distance measures are based on the current number of clusters against the previous number of clusters
